# Measuring Accessibility to Healthcare Using Taxi Trajectories Data: A Case Study of Acute Myocardial Infarction Cases in Beijing

**DOI:** 10.34172/ijhpm.2022.6653

**Published:** 2022-09-26

**Authors:** Yuwei Su, Zhengying Liu, Jie Chang, Qiuju Deng, Yuyang Zhang, Jing Liu, Ying Long

**Affiliations:** ^1^School of Architecture, Tsinghua University, Beijing, China.; ^2^School of Urban Design, Wuhan University, Wuhan, China.; ^3^Department of Epidemiology, Beijing An Zhen Hospital, Capital Medical University, Beijing Institute of Heart, Lung and Blood Vessel Diseases, The Key Laboratory of Remodeling-Related Cardiovascular Diseases, Ministry of Education, Beijing, China.; ^4^Beijing Municipal Key Laboratory of Clinical Epidemiology, Beijing, China.; ^5^School of Architecture and Hang Lung Center for Real Estate, Key Laboratory of Eco Planning & Green Building, Ministry of Education, Tsinghua University, Beijing, China.

**Keywords:** Healthcare Accessibility, Taxi GPS Traces, Retrospective Measurement, Beijing

## Abstract

Several methods have been applied to measure healthcare accessibility, ie, the Euclidean distance, the network distance, and the transport time based on speed limits. However, these methods generally produce less accurate estimates than actual measurements. This research proposed a method to estimate historical healthcare accessibility more accurately by using taxi Global Positioning System (GPS) traces. The proposed method’s advantages were evaluated vis a case study using acute myocardial infarction (AMI) cases in Beijing in 2008. Comparative analyses of the new measure and three conventionally used measures suggested that the median estimated transport time to the closest hospital with percutaneous coronary intervention (PCI) capability for AMI patients was 5.72 minutes by the taxi GPS trace-based measure, 2.42 minutes by the network distance-based measure, 2.28 minutes by the speed limit-based measure, 1.73 minutes by the Euclidean distance-based measure; and the estimated proportion of patients who lived within 5 minutes of a PCI-capable hospital was 38.17%, 89.20%, 92.52%, 95.05%, respectively. The three conventionally used measures underestimated the travel time cost and overestimated the percentage of patients with timely access to healthcare facilities. In addition, the new measure more accurately identifies the areas with low or high access to healthcare facilities. The taxi GPS trace-based accessibility measure provides a promising start for more accurately estimating accessibility to healthcare facilities, increasing the use of medical records in studying the effects of historical healthcare accessibility on health outcomes, and evaluating how accessibility to healthcare changes over time.

## Background

 Healthcare accessibility has been intensively studied in the field of public health not only for policy evaluation purposes^1^ but also as a factor explaining many health outcomes, such as colorectal cancer survival^2^ and acute myocardial infarction (AMI) and stroke mortality.^3^ While decades of attention have been given to healthcare accessibility, many questions remain unexplored. For example, how does individual-based healthcare accessibility change over time? How does healthcare accessibility for individuals in the past relate to diseases in the past? In addition, while much is known about the relationship between healthcare accessibility and various kinds of health outcomes, most of the accumulated evidence to date has been drawn from cross-sectional. Compared to cross-sectional studies, cohort studies are better for assessing causality. However, a prospective study is usually very expensive and time-consuming. A retrospective cohort study is relatively cheap and easy and quick to conduct. If we want to find an association between healthcare accessibility and health outcomes in a retrospective cohort study, the key is to measure accessibility to healthcare in the past. Huge amounts of paper and electronic medical records in the past that included patient demographics, places of residence, diagnoses, disease statuses, etc. are potentially valuable sources of data for addressing the abovementioned questions. Data from patients’ electronic medical records had been used in some studies in different countries and was proven to be available for scientific research.^4-9^ However, historical medical records are greatly underused in healthcare accessibility studies. One reason is due to geoprivacy issue which might hinder using historical medical record data. Another reason might be that places of residence and the locations of healthcare facilities in historical medical records were described using text, and earlier geocoding technology could not easily and quickly transform many text-based locations to geographic coordinates with high accuracy to support healthcare accessibility analysis. This case is particularly apparent for Chinese text-based locations because Chinese text, unlike English, has no delimiters between words, and it is highly difficult to string a phrase and extract accurate address elements to match a reference database.^10^ However, with recent advancements in geocoding technology, it has become technically feasible to transform many text-based locations into geographic coordinates.

 After data are geocoded, the key question that remains is how to accurately measure historical healthcare accessibility. In the literature, various measures have been developed to evaluate individual-based healthcare accessibility. Table S1 of Supplementary file 1 summarizes the three most used measures of healthcare accessibility, which are the following: (1) the Euclidean (straight-line) distance to the nearest healthcare facility, (2) the network distance to the nearest healthcare facility, and (3) the network transport time to the nearest healthcare facility based on the speed limits across different road types. There are also some other dynamic transport network-based measures such as the network transport time to the closest healthcare facility estimated by Google Maps API.^11^ However, as this study mainly focuses on the measure of historical healthcare accessibility and those measures are only available to estimate real-time healthcare accessibility instead of historical healthcare accessibility, they are not considered in this study. The Euclidean distance to the closest healthcare facility is the length of the straight geometric line linking a patient’s residential location and the nearest healthcare facility around the patient. The procedure of computing Euclidean distances requires only geographic location data related to the patient residences and the healthcare facility and thus has the advantage of simplicity. Many studies have used Euclidean distances as measures of the geographic accessibility of healthcare.^12^ However, such a measure is somewhat inaccurate as patients follow the layout of the road network and not a straight line to access healthcare. Moreover, when measuring healthcare accessibility for patients in different time periods, the Euclidean distance measure cannot capture healthcare accessibility changes over years due to the impacts of factors such as the expansion of road networks and the dynamics of traffic conditions in different years and thus could lead to a lack of precision.

 The network distance to the closest healthcare facility is measured by the length of the shortest road network linking a patient’s residential location and the nearest healthcare facility to the patient. The calculation of the network distance to the closest healthcare facility requires data on both geographic locations related to patients’ residences and healthcare facilities and road networks. The network distance is considered to be superior to the Euclidean distance as it allows to measure the lengths of actual pathways between two sites.^13^ Many studies have used network distances as measures of accessibility to healthcare from individuals’ residential locations.^14,15^ While this approach offers greater precision than the Euclidean distance, a major inadequacy of such a measure is that it fails to account for travel impedances such as speed limits. Another disadvantage of the network distance measure is that when measuring healthcare accessibility for patients in different time periods, it fails to capture healthcare accessibility changes resulting from the dynamics of traffic conditions over years, although healthcare accessibility changes over years due to the expansion of road networks could be captured.

 Regardless of which of the abovementioned methods is used to obtain the distances, distance alone does not fully explain accessibility since travel impedances like speed limits are also influential.^16^ Since speed limits can be taken into account, travel time based on the speed limits on each type of road has been considered as a better indicator of accessibility.^17,18^ Travel time estimates based on speed limits have been extensively applied in evaluating geographic accessibility to healthcare.^1^ The computation of the travel time necessitates data on geographic locations related to patients’ residences and healthcare facilities and road networks with speed limits available for each road segment. However, this accessibility measure is also generally inaccurate because it is estimated based on speed limits, which cannot represent actual travel speeds. More importantly, when measuring healthcare accessibility for patients over different time periods, travel time measures based on speed limits cannot capture the temporal variation in healthcare accessibility over different years if the speed limits remain unchanged, but the actual travel speed has been declining due to an increase in traffic volume caused by the increase in the number of private vehicles.

 In summary, there is a lack of a more accurate method for evaluating historical healthcare accessibility in the current literature. The objectives of this study are to propose a new method to estimate historical healthcare accessibility more accurately and to illustrate the proposed method’s advantages. Briefly, the new accessibility measure is computed as network distances divided by actual travel speeds across the road network estimated using taxi Global Positioning System (GPS) traces. In comparison with the three conventional methods, this method is more accurate because it considers actual traffic delays and road traffic speeds estimated using taxi GPS traces. For illustration, we applied the new method to estimate accessibility to hospitals with percutaneous coronary intervention (PCI) capacity across patients with AMI in Beijing, China in 2008 and evaluated the errors of three conventional accessibility measures by comparing them to the most accurate accessibility measure.

## Methods

 In this section, we firstly described how to use the taxi GPS trace data in combination with the geographic location data related to patients’ residences and healthcare facilities and road network data to estimate historical healthcare accessibility, and then presented data collection.

###  Geocode

 The process of taxi-based healthcare accessibility estimation for patients at a certain time begins with geocoding using a batch geocode tool and ArcGIS. Geocoding is a process of converting text, such as the address of a hospital or a patient’s residential location, to geographic coordinates. Since the resulting locations are geographic features with attributes, therefore they can be applied for mapping or spatial analysis. As many historical medical records are paper-based and there are shortcomings in terms of their accuracy and completeness,^19^ the challenge here is how to convert thousands of residential addresses into coordinates and display them on a map with a high level of accuracy. The proposed solution is to use a batch geocode tool in combination with manual correction. For example, the tool “DataMap for Excel” could be applied. This tool can automatically convert a list of text-based addresses input from a table into GCJ-02 coordinates, which can be further transformed into WGS-84 coordinates by GeoSharp.

###  Aggregate Patients Into a 500 m × 500 m Grid

 Considering the fact that detailed individual-level data might not be available for many countries in the world where privacy regulations are strict, the georeferenced data can be transformed into a grid structure and aggregated into grid cells at a certain spatial resolution such as 500 m × 500 m to guarantee the protection of privacy of individuals.

###  Estimate Congested Travel Speeds Using Taxi GPS Traces Data

 Third, congested travel speeds were estimated using taxi GPS traces. As only low-sampling frequency traces are available in most cases for operational and data protection reasons, there is a challenge map-matching a continuous space GPS trajectory to the road network and accurately inferring the path travelled between two observations. Moreover, the availability of detailed road networks is usually a restriction because such datasets are usually expensive to access, build or hold by state-controlled organizations. Therefore, network representation is also a challenge. See Deng et al^20^ for more details of how to address the two key challenges.

###  Build the Transport Network Dataset in ArcCatalog

 Fourth, a network dataset that considers many network elements, such as congested travel speeds, turn impedances, and one/two-way streets, is created in ArcCatalog.

###  Compute the Healthcare Accessibility in ArcMap

 Finally, with the ArcGIS Network Analyst extension, the accessibility to the nearest healthcare for patients at a certain time can be calculated. Specifically, in ArcMap, both the Network Analyst extension and the OD cost matrix function are activated. After the predefined network dataset is loaded to the active project, it is important to specify several network parameters such as impedance, distance unit, etc.

###  Data Sources

 Patients with AMI were selected for this case study to illustrate the advantage of taxi GPS trace-based healthcare accessibility measure over three conventional methods because the likelihood of favourable outcomes after reperfusion therapy largely depends on accessibility to a PCI hospital.

 We obtained a total of 8807 cases of AMI among permanent residents within the Fifth Ring Road of Beijing from the Beijing Monitoring System for Cardiovascular Diseases from January 1, 2008 to December 31, 2008. The spatial distributions of AMI cases and permanent residents are presented in Figure S1 (see Supplementary file 1). The data for the year 2008 was used because it nearly represents the earliest year since which GPS tracking devices have been installed on taxis of many cities in China and one aim of the current study was to measure historical healthcare accessibility. Details of the system have been reported previously.^21^ The residential address of each case of AMI and the addresses of all PCI-capable hospitals were identified in the monitoring system. Specific geographic coordinates (longitudes and latitudes) for all cases of AMI and PCI-capable hospitals were acquired according to the method described in the above section. In this study, the georeferenced data have been transformed into a grid structure and the number of patients were aggregated in 500 m × 500 m grid cells to guarantee the protection of privacy of individuals. The resolution of a 500 m × 500 m grid cell was selected for three reasons. First, a grid cell should have a certain number of patients to avoid leaking their confidential information, however, a higher resolution grid is easy to reveal privacy information. As shown in Table S2, when a 100 m × 100 m grid is adopted, the study area is divided into 67 190 grids, among which 5767 grids contain cases and 3941 contain 1 case accounting for 68.34% of the grids with cases. Among the grids with cases, the average number of cases in each grid is 2, and the median number is 1. However, when a 500 m × 500 m grid is adopted, the study area is divided into 1575 grids, among which 1553 grids contain cases and 325 contain 1 case accounting for 20.93% of the grids with cases. Among the grids with cases, the average number of cases in each grid is 6, and the median number is 4. Second, a higher resolution grid also increases the time of data processing. Especially when the choice of a 500 m × 500 m grid has revealed the accuracy of the taxi GPS trace-based accessibility measure, the gains using a higher resolution grid such as a 100 m × 100 m grid cannot make up the losses. Third, a lower resolution grid moderates the advantage of individual-level data in measuring healthcare accessibility with high accuracy. As shown in Figure S2, when a 1000 m × 1000 m grid is adopted, the results indicated that some patients had access to a PCI-capable hospital within a 5 to 10 minutes period, however, they actually had access to the hospital within a 10 to 15 minutes period which was revealed at the spatial resolution of 500 m × 500 m.

 The road network data of Beijing in 2008 were provided by Deng et al.^20^ The GPS trajectory dataset employed in this study includes 1 500 000 observations from 10 357 taxis within the Fifth Ring Road of Beijing covering the 5:00-11:00 am period from Monday the 4th to Wednesday the 6th of February 2008. The data were derived from Microsoft Research Asia. Each observation contains details of the taxi identifiers, locations, and timestamps. We did not calculate the congested link speeds from the taxi GPS traces; instead, we directly obtained the link speed data within Beijing’s Fifth Ring Road from Deng et al^20^ who proposed the method employed in this study for calculating congested link speeds from taxi GPS traces. The area within the Fifth Ring Road of Beijing is the area from the centre of Beijing to the Fifth Ring Road in Beijing, which is the main area of traffic congestion. This area includes 7 main urban areas of Beijing.

 The speed limit data were obtained from the code for the design of urban road engineering.^22^

## Results

###  Median Travel Time Estimated by Four Types of Accessibility Measures

 Figure S3 presents the distributions of the estimated travel time by four accessibility measures through histograms. Since they are skewed, we used the median which is more representative of the average. Table shows the results for the median healthcare accessibility across the four types of accessibility measures for patients in 2008. Note that the Euclidean distance and network distance are measures of distance while travel time is a measurement of time. For the sake of comparison, there is a need for converting time and distance. Therefore, the concept of speed, a simple ratio between distance and time, was introduced. This study calculated the average travel speed as the mean of the speed limits across different road types over the city, yielding the following value: the average distance travelled in one hour is 46.25 km. Based on the average speed, Euclidean and network distances were then transformed into travel times. The results indicated that the median travel times to the nearest hospital with PCI capacity estimated with the taxi GPS trace-based measure was 5.72 minutes. Compared with the newly proposed measure, the network distance-based measure mis-estimated the travel time to the closest PCI-capable hospital by 3.30 minutes, the speed limit-based measure mis-estimated it by 3.44 minutes, and the Euclidean distance-based measure mis-estimated it by 3.99 minutes. A scatter plot showing the relationship between the pairs of measures is presented in Figure S4, which further confirmed that the network distance-based measure was closer to the taxi GPS trace-based measure than the speed limit-based measure, followed by the Euclidean distance-based measure.

**Table:** Estimated Healthcare Accessibility (min) in 2008 by Different Metrics

	**Driving Times to the Closest PCI-Capable Hospital in 2008**			
	**Taxi GPS Traces**	**Network Distance**	**Speed Limits**	**Euclidean Distance**
Median	5.72	2.42	2.28	1.73
Mean	6.32	2.82	2.56	2.08
SD	3.27	1.78	1.48	1.44

Abbreviations: PCI, percutaneous coronary intervention; SD, standard deviation.

 Then, we compared the standard deviations of the estimated travel times across the four accessibility measures. The results showed that the standard deviation of the travel time computed using the taxi GPS trace-based measure was 3.27 minutes. In comparison with the new measure, the travel times estimated by the other three measures displayed a lower standard deviation with values of 1.78 minutes, 1.48 minutes, and 1.44 minutes, respectively. This result means that the conventional accessibility measures underestimated the variations of healthcare accessibility across individuals.

###  Proportion of Patients Whose Travel Time Was Within 5 minutes

 Differences in measures of the proportion of patients who have timely access to a PCI-capable hospital depend on not only differences in the accuracy of measurement methods but also the actual proportion. Suppose that 90% of the people in a city lived within 60 minutes of a PCI-capable hospital. All the proportions estimated using the four accessibility measures would be more than 90% as these four measures consider fewer travel impedances than the actual measure. Then, the differences in the measures of the proportion are less than 10% regardless of the differences in the accuracies of the four measurement methods. In other words, the differences in the accuracy of measurement methods could not be significantly reflected by the differences in the proportions. Here, we compared the four accessibility measures mainly based on the proportion of patients who had access to a PCI-capable hospital within a 5-min period instead of other time periods because it can significantly reflect the differences in the accuracy of these four accessibility measures.

**Figure 1:**
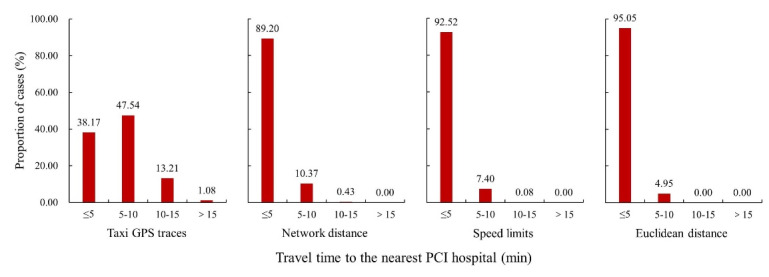
Distributions of the Estimated Travel Times to the Nearest PCI-Capable Hospital for AMI Patients in 2008 by Different Accessibility Measures. Abbreviations: GPS, Global Positioning System; PCI, percutaneous coronary intervention; AMI, acute myocardial infarction.

 The differences in the proportions of patients who lived within 5 minutes of a PCI-capable hospital are presented in Figure 1. The results estimated using the taxi GPS trace-based measure indicated that 38.17% of the patients on average had access to a PCI-capable hospital within 5 min. In comparison with the new measure, the speed limit-based measure overestimated the proportion of patients who lived within 5 minutes of a PCI-capable hospital by 51.03%, the network distance-based measure overestimated it by 54.35%, and the Euclidean distance-based measure overestimated it by 56.88%.

###  Spatial Distributions of the Estimated Travel Times to the Nearest PCI-Capable Hospital for AMI Patients in 2008 by Accessibility Measure

**Figure 2:**
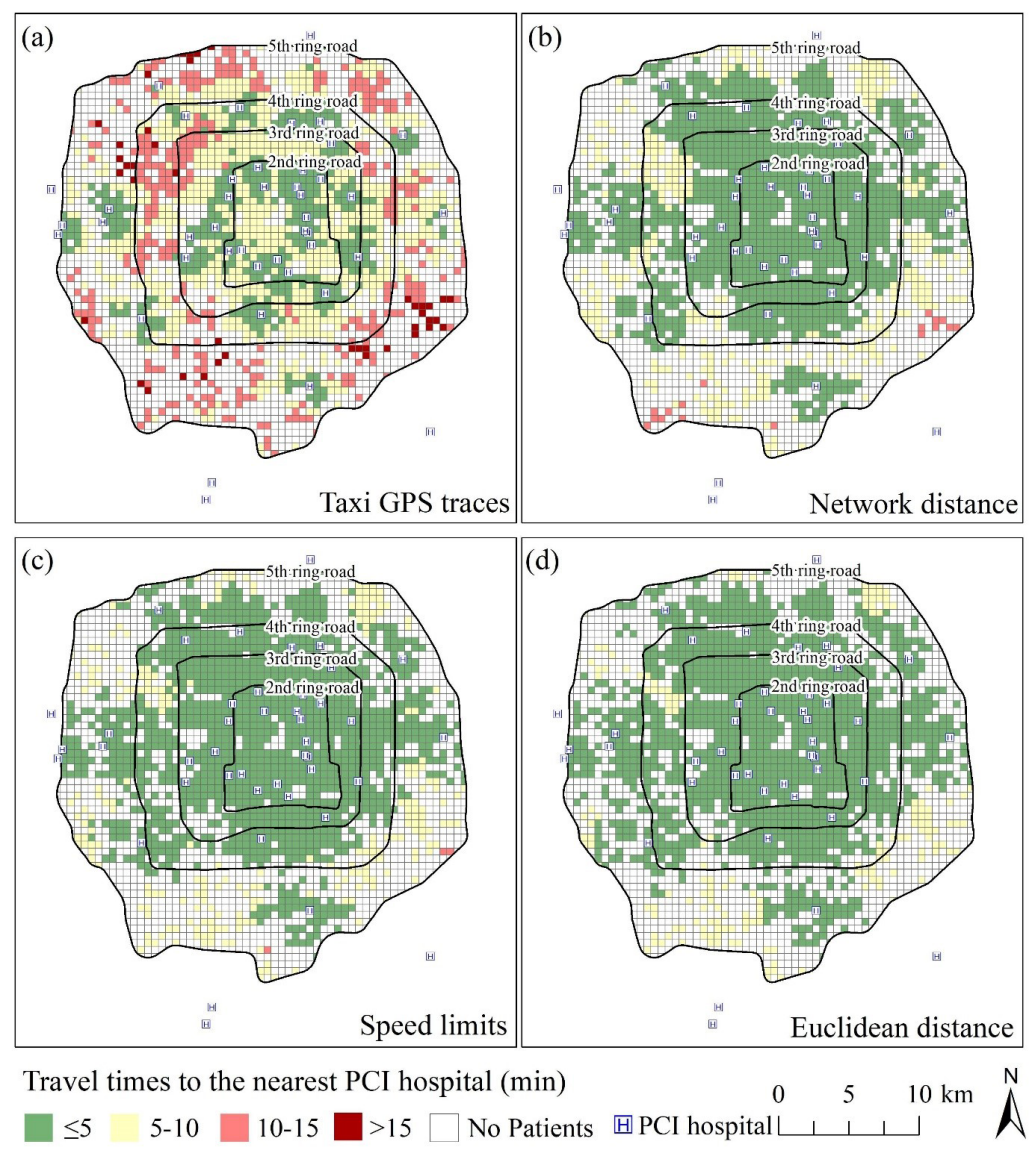
Spatial Distributions of the Estimated Travel Times to the Nearest PCI-Capable Hospital for AMI Patients in 2008 by Different Accessibility Measures. Abbreviations: GPS, Global Positioning System; PCI, percutaneous coronary intervention; AMI, acute myocardial infarction.

 The spatial distribution of the transport time to the closest PCI-capable hospital for patients in 2008 estimated using the taxi GPS trace-based measure was compared to those computed using three conventional accessibility measures (Figure 2). Figure 2a shows the spatial distribution of the transport time among these patients to the closest PCI-capable hospital estimated using the new proposed method. The result indicated that a great number of patients who lived in the areas located between the fourth and fifth Ring Roads had an estimated transport time of more than 10 minutes to the nearest PCI-capable hospital. However, Figure 2b, 2c and 2d show that none or only a small number of patients in these areas did not live within 10 minutes of a PCI-capable hospital. Normally, we should focus on patients with a transport time of larger than 60 minutes to the nearest PCI-capable hospital because hospital arrival within a 60-minute period maximizes the likelihood of favourable outcomes after reperfusion therapy,^23^ and those living outside this time period for a PCI-capable hospital should be given a special focus. However, the overall number and geographic distribution of PCI-capable hospitals appears sufficient for Beijing, the capital of China, especially for the area inside the fifth Ring Road and all people lived within 60 minutes of a PCI-capable hospital. Therefore, we here specifically focused on patients with a transport time of greater than 10 minutes to the nearest PCI-capable hospital just to illustrate the accuracy of the taxi GPS trace-based accessibility measure compared to other three traditional measures.

## Discussion

 This study proposed a taxi GPS trace-based healthcare accessibility measure and contrasted it with three commonly used accessibility measures, ie, speed limit-based, network distance-based, and Euclidean distance-based, in the context of PCI-capable hospital accessibility analysis for patients with AMI in Beijing in 2008.

 The results indicated that the median travel time estimated by the taxi GPS trace-based method to the nearest PCI-capable hospital for patients with AMI in Beijing in 2008 was 5.72 minutes. However, the three conventional accessibility measures underestimated the travel time. In emergency responses, where the results are sensitive to even small variations in transport time to healthcare facilities,^24^ an accurate measure of healthcare accessibility is essential. Therefore, it is recommended that future studies employ the new proposed accessibility measure that minimizes measurement errors in healthcare accessibility analysis.

 The findings of our study showed that 38.17% of the patients in Beijing lived within 5 min of a PCI-capable hospital. In comparison with taxi GPS trace-based accessibility measures, the three conventionally used accessibility measures overestimated the proportion of patients who had timely access to PCI-capable hospitals. The proportion of people with timely access to healthcare in an area is important information for policymakers to determine the extent to which an effective intervention effort is needed to improve access to healthcare. From this perspective, we argue that estimating healthcare accessibility using taxi GPS traces will help health policymakers make more informed and precise decisions.

 Based on the taxi GPS trace-based accessibility measure, we found that many patients lived in the areas located between the fourth and fifth Ring Roads where the time required to reach a PCI-capable hospital by car was more than 10 minutes. However, the results based on three conventionally used methods indicated that none or only a small number of patients in these areas travelled more than 10 minutes to access a PCI-capable hospital. This is misleading. Precisely identifying where people who have a particularly high estimated transport time to the nearest healthcare facilities reside can inform policymakers about where interventions to enhance physical access to healthcare facilities are required most urgently.^25^ Therefore, regarding informing the implementation of policy interventions, identifying areas with lower accessibility in the past should be based on the taxi GPS trace-based accessibility measure, which is more accurate than the other measures.

 This study has several limitations. First, the proposed method is only applicable to estimating the accessibility of healthcare facilities based on taxis or private cars instead of ambulance-based accessibility. However, this did not weaken the value of this study, since a lot of AMI patients use taxis or private cars to transport themselves to hospitals instead of emergency medical services (EMS). Regarding the low use of EMS, Sui et al^26^ argued that it is because people lack knowledge on recognizing and responding to potential AMI symptoms. For patients in countries such as Mumbai and India, they usually do not use EMS because no enough EMS are available.^27^ Second, we did not possess data on the actual observed travel times and just performed a preliminary validation by comparing these four accessibility metrics. Although our assumption that the taxi GPS trace-based travel time measurement is a more accurate estimate than the other three traditional metrics is with well-understood theoretical foundations, future studies are needed to validate the proposed method by examining the extent to which these four estimated travel times are different from the observed travel time respectively. Third, we did not obtain the data on the actual PCI-capable hospitals which the patients choose. Instead, we used the nearest PCI-capable hospitals to examine the accessibility of the patients’ home addresses, which may be problematic, because patients may not choose the nearest hospital in reality. However, it is a matter of indifference, given that the purpose of this study was to illustrate the proposed method’s advantages by comparing the proposed method’s estimated results with those computed by three conventionally used methods. Since the proposed method’s advantages have been demonstrated under the circumstances that healthcare accessibility was measured based on patients’ home addresses and the nearest healthcare, its advantages would be more obvious when healthcare accessibility was measured based on patients’ home addresses and the actual healthcare that they choose, because the errors in accessibility estimates based on traditional methods became larger than that based on the proposed method as the true distances between the locations of the patients’ residence and of healthcare facilities increased. Finally, this study assumed that the residential location of patients could represent the address location where they were taken to hospital. Although this assumption may be valid for older adults who often spend a large proportion of their time inside their house, patients’ residential address does not always reflect the place where they were taken to hospital, especially for adults who work full time. Therefore, it should be noted that using the actual place where patients have onset is more effective when examining the relationship between healthcare accessibility and mortality of certain diseases.

## Conclusion

 This research proposes a new accessibility measure based on taxi GPS trace data. By comparing the approach with three commonly used accessibility measures, several advantages are identified: the new measure more accurately estimates the healthcare accessibility of individuals, estimates the proportion of patients who have timely access to healthcare facilities, and identifies the areas with low or high access to healthcare facilities. The method developed based on taxi GPS traces provides a promising start and opens new opportunities for more accurately estimating accessibility to healthcare facilities, increasing the use of medical records in studying the effects of historical healthcare accessibility on health outcomes, and evaluating how accessibility to healthcare changes over time.

 Acknowledgements

 The authors would like to express special thanks to Steve Denman, Debbie Deng and Ying Jin of University of Cambridge, United Kingdom for providing the road network data with GPS traces for this study.

## Ethical issues

 The study was approved by the Ethics Committee at the Beijing An Zhen Hospital (ks2020010).

## Competing interests

 Authors declare that they have no competing interests.

## Authors’ contributions

 YS and ZL jointly designed the study, YS conducted all data analysis, ZL wrote first and all drafts of manuscript. JC, QD, and YZ provided critical feedback and helped improve the research, analysis and manuscript. JL and YL supervised the study. All authors read and approved the final manuscript.

## Funding

 This work was supported by the Pathways to Equitable Healthy Cities grant from the Wellcome Trust (209376/Z/17/Z), the National Natural Science Foundation of China (82073635), the Beijing Nova Programme Interdisciplinary Cooperation Project (Z191100001119017) and the Capital’s Funds for Health Improvement and Research (2020-1-1051). For the purpose of Open Access, the author has applied a CC BY public copyright licence to any Author Accepted Manuscript version arising from this submission.

## Supplementary files


Supplementary file 1 contains Tables S1-S2 and Figures S1-S4.
