# Supplementary material for: Measuring Accessibility to Healthcare Using Taxi Trajectories Data: A Case Study of Acute Myocardial Infarction Cases in Beijing
Source: Int J Health Policy Manag. 2022 Sep 26;12:6653. doi: 10.34172/ijhpm.2022.6653 (PMC10125134; doi:10.34172/ijhpm.2022.6653)
Supplement: Supplementary file 1 — contains Tables S1-S2 and Figures S1-S4. [file ijhpm-12-6653-s001.pdf]

Article title: Measuring Accessibility to Healthcare Using Taxi Trajectories Data: A Case Study of Acute Myocardial Infarction Cases in Beijing

Journal name: International Journal of Health Policy and Management (IJHPM)

Authors' information: Yuwei Su[1,2], Zhengying Liu[1], Jie Chang[3,4], Qiuju Deng[3,4], Yuyang Zhang[1], Jing Liu[3,4]*, Ying Long[5]*

[1]School of Architecture, Tsinghua University, Beijing, China.

[2]School of Urban Design, Wuhan University, Wuhan, China.

[3]Department of Epidemiology, Beijing An Zhen Hospital, Capital Medical University, Beijing Institute of Heart, Lung and Blood Vessel Diseases, The Key Laboratory of Remodeling-Related Cardiovascular Diseases, Ministry of Education, Beijing, China.

[4]Beijing Municipal Key Laboratory of Clinical Epidemiology, Beijing, China.

[5]School of Architecture and Hang Lung Center for Real Estate, Key Laboratory of Eco Planning & Green Building, Ministry of Education, Tsinghua University, Beijing, China.

¶Both authors contributed equally to this paper.

(*Corresponding author: Ying Long; Email: ylong@tsinghua.edu.cn & Jing Liu; Email: jingliu@ccmu.edu.cn)

Supplementary file 1

Table S1. A summary of the measures of individual-based health care accessibility

Measures	Measurement unit	Required data	Travel impedances considered	Capturing accessibility dynamics over years [a]
Euclidean (straight-line) distance to the nearest health care facility	Meters	Geographic data	Euclidean distance	No
Network distance to the nearest health care facility	Meters	Geographic data and road network data	Network distance	Yes, but partially
Network travel time to the nearest health care facility based on the speed limits across different road types	Minutes	Geographic data, road network data, and speed limit data	Network distance and speed limits	Yes, but partially

Note: [a] Suppose that the locations of residences and health care facilities remain unchanged.

Table S2. The distribution of patients per cell at each

Resolution	No. of grids	No. of grids with cases	No. of grids with 1 case	No. of cased in each grid	
				Mean	Median
100m × 100m	67,190	5,767	3,941	2	1
500m × 500m	1,575	1,553	325	6	4
1,000m × 1,000m	732	579	72	15	10

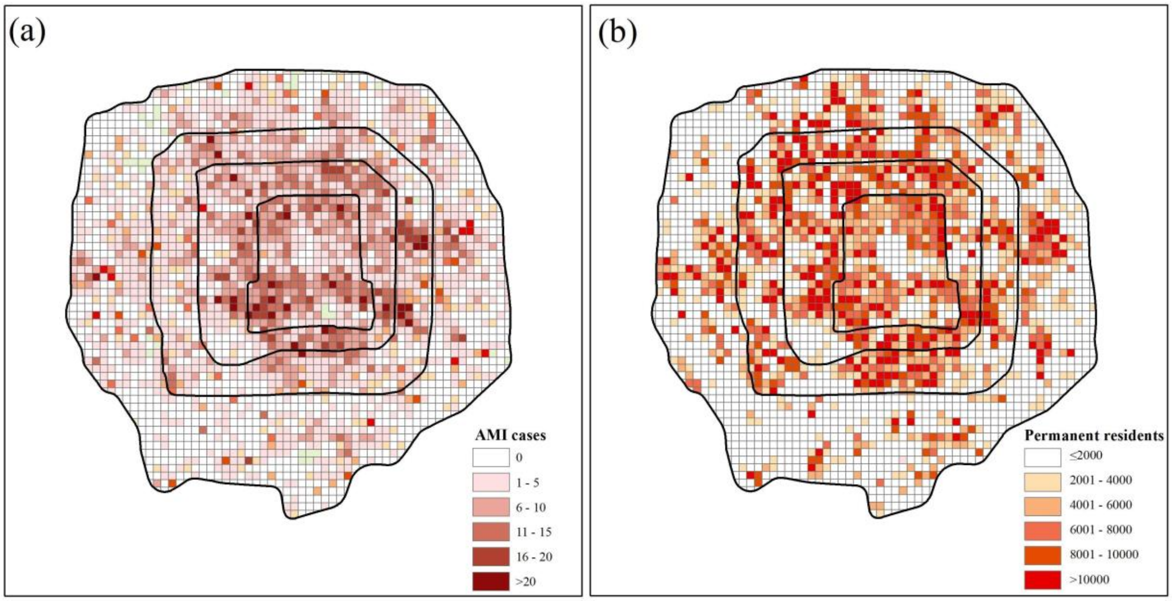

Figure S1. Spatial distributions of AMI cases and permanent residents

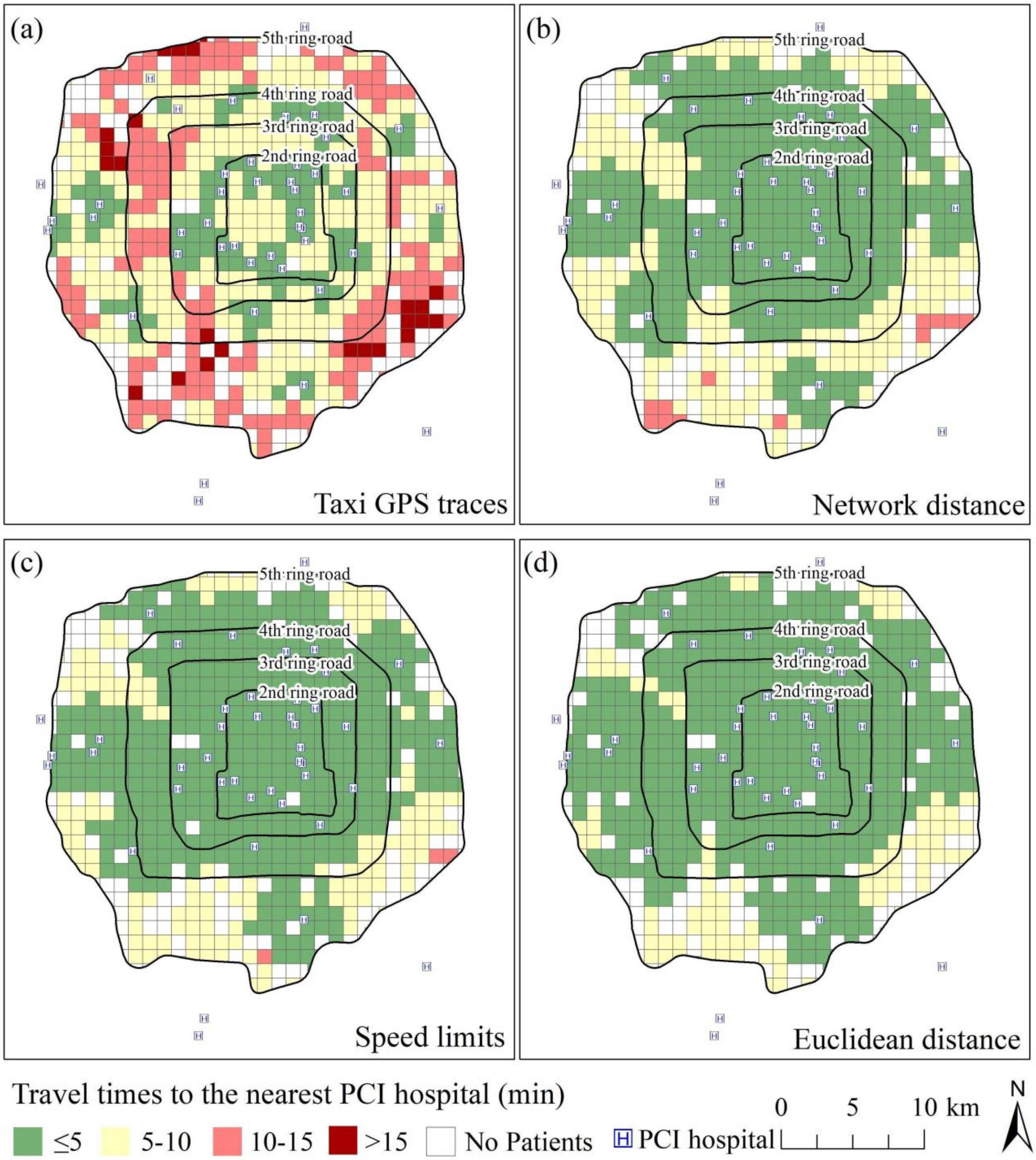

Figure S2. Spatial distributions of the estimated travel times to the nearest PCI-capable hospital for AMI patients in 2008 by different accessibility measures at the spatial resolution of 1,000 m × 1,000 m

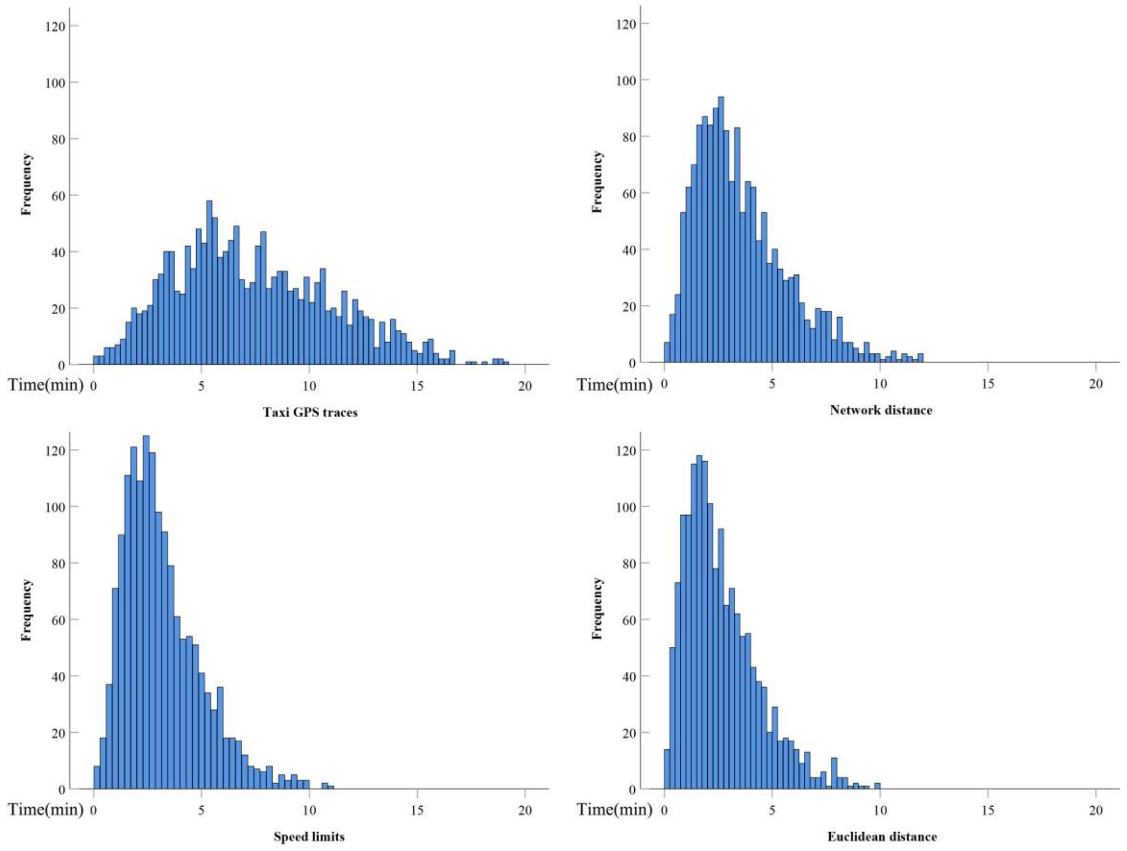

Figure S3. Distributions of estimated travel time by four accessibility measures

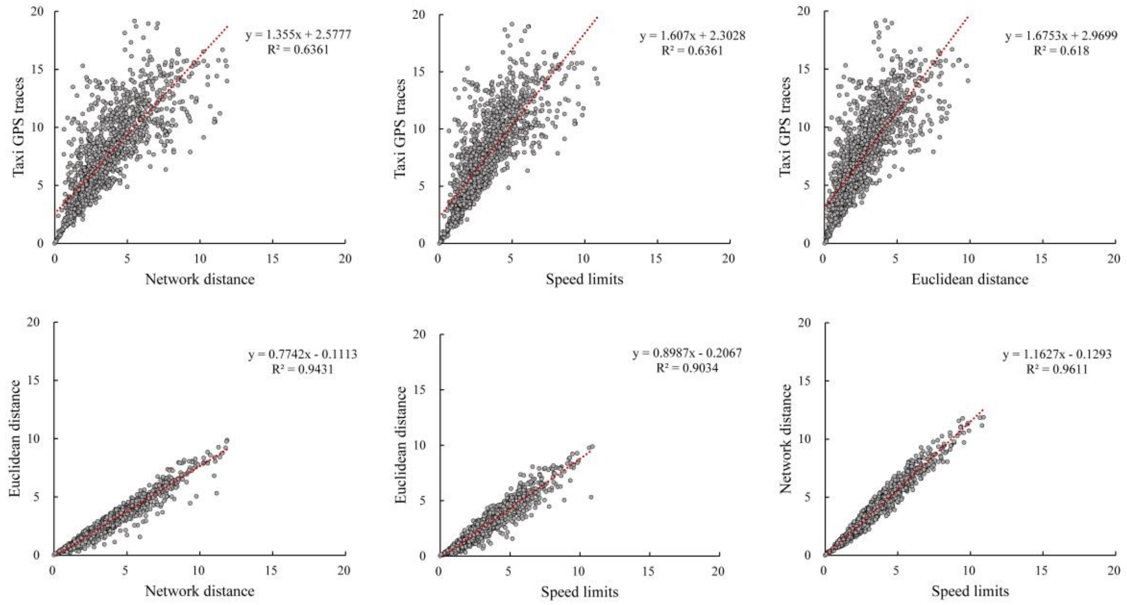

Figure S4: Scatter plot of any two accessibility measures
